# Effects of Cytokines (or Activating Factors) on Arterial Endothelial Cells

**DOI:** 10.3390/ijms26178142

**Published:** 2025-08-22

**Authors:** Leon M. T. Dicks

**Affiliations:** Department of Microbiology, Stellenbosch University, Private Bag X1, Matieland, Stellenbosch 7602, South Africa; lmtd@sun.ac.za

**Keywords:** internal mammary arteries, coronary arteries, anatomical and physiological differences

## Abstract

The internal mammary arteries (IMAs) and coronary arteries share many common characteristics. The inner layer (tunica intima, or intima) of both arteries is lined with a smooth, longitudinally orientated monolayer of endothelial cells (ECs), connective tissue, and an internal elastic lamina that separates the tunica intima from the tunica media (middle layer). The intima of IMAs is lined with an additional protective layer, the neointima, containing vascular smooth muscle cells (VSMCs). The neointima, located between the intima and internal elastic lamina, protects IMAs from damage by assisting in the remodeling of VSMCs. Coarse longitudinal folds in the internal elastic lamina of IMAs partially prevent the infiltration of VSMCs into damaged IMAs, and intimal thickening is thus less likely to occur. Inflamed IMAs resist the migration of monocytes across the endothelial layer and prevent the formation of lipid-rich macrophages (foam cells) within the subintimal or medial layers of arteries. IMAs are thus less likely to form plaques and develop atherosclerosis (AS). Higher levels of prostacyclin (PGI2) in IMAs prevent blood clotting. The anti-thrombotic agents, and production of tumor necrosis factor α (TNF-α), interferon-γ (INF-γ), and visfatin render IMAs more resistant to inflammation. An increase in the production of nitric oxide (NO) by ECs of IMAs may be due to small ubiquitin-like modifier (SUMO) proteins that alter the nuclear factor kappa B (NF-κB) and TLR pathways. The production of reactive oxygen species (ROS) in IMAs is suppressed due to the inhibition of NADPH oxidase (NOX) by a pigment epithelium-derived factor (PEDF), which is a serine protease inhibitor (SERPIN). In this review, a comparison is drawn between the anatomy of IMAs and coronary arteries, with an emphasis on how ECs of IMAs react to immunological changes, rendering them more suited for coronary artery bypass grafts (CABGs). This narrative review covers the most recent findings published in PubMed and Crossref databases.

## 1. Introduction

**Figure 1:**
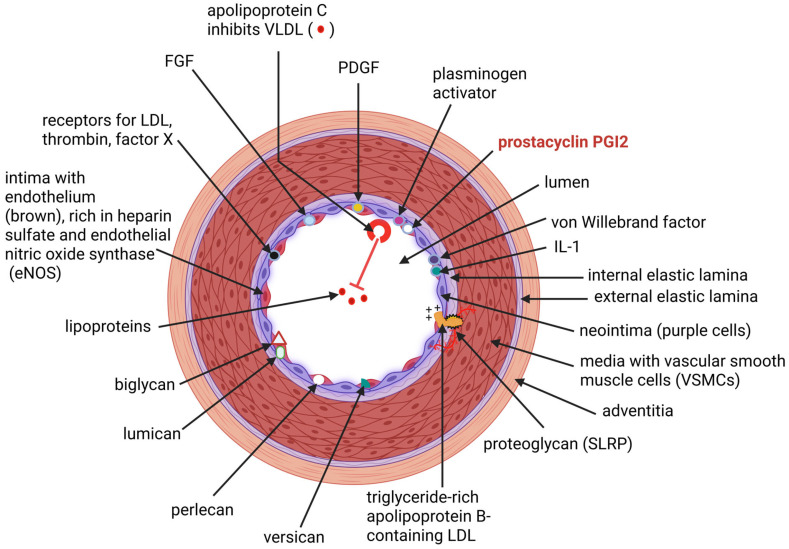
Schematic representation of an internal mammary artery (IMA), consisting of three well-defined concentric layers: the intima (tunica intima), the media (tunica media), and an outer (external) layer, the adventitia. The intima comprises a single layer of longitudinally orientated endothelial cells (ECs), connective tissue, and an internal elastic lamina. The neointima is located between the intima and the internal elastic lamina and is composed of smooth muscle cells. The endothelium of IMAs contains prostacyclin (PGI2), von Willebrand factor, interleukin-1 (IL-1), plasminogen activator, platelet-derived growth factor (PDGF), and fibroblast growth factor (FGF). The endothelium also contains low-density lipoprotein (LDL), thrombin, factor X, small leucine-rich proteoglycans (SLRPs), e.g., biglycan and lumican, and large proteoglycans (versican and perlecan.) The production of PG12 is higher in IMAs compared to coronary arteries. The schematic representation was constructed using Biorender, (Created in Biorender. L.M.T. Dicks. 2025. https://app.biorender.com/illustrations/689b2c9202cb36d3d34c1fbe accessed on 20 August 2025).

Internal mammary arteries (IMAs) and coronary arteries share many structural and physiological properties. Both arteries are composed of three concentric layers, i.e., an inner (luminal) layer, the intima (tunica intima); a middle layer, the media (tunica media); and an outer (external) layer, the adventitia. The intima of coronary arteries is lined with smooth, longitudinally orientated endothelial cells (ECs), connective tissue, and an internal elastic lamina. Although the intima of IMAs is similar in composition, it lacks a prominent internal elastic lamina. Instead, the intima of IMAs has an additional protective layer, the neointima, which contains vascular smooth muscle cells (VSMCs) and is separated from the media by an internal non-fragmented (and less defined) internal elastic lamina (Figure 1) [1]. The neointima protects IMAs from damage by assisting in the remodeling of VSMCs, i.e., migration, proliferation, and phenotypic modulation, which helps repair vessel injuries and maintain healthy blood flow [1]. The endothelium of IMAs has fewer small openings and lower intercellular junction permeability compared to other arteries [1]. This suppresses the passage of lipoproteins and other molecules that may contribute to plaque formation and partially prevents the migration of VSMCs from the tunica media to the intima [1]. IMAs are thus less prone to intimal thickening, a precursor of atherosclerotic plaque [2,3]. The internal elastic lamina in IMAs has a higher fold index (a measure of folding) compared to other arteries, such as the left anterior descending artery (LAD). A higher fold index (more folds in the lamina) is associated with a lower chance of developing atherosclerosis (AS) [1]. These properties render IMAs more suitable as conduits in coronary artery bypass graft (CABG) surgery.

The endothelium of IMAs contains prostacyclin (PGI2) that prevents blood clotting, von Willebrand factor that attaches platelets to the arterial wall, interleukin-1 (IL-1) that responds to inflammation, plasminogen activator that dissolves blood clots, platelet-derived growth factor (PDGF) that controls cell growth and repairs cellular damage, and fibroblast growth factor (FGF) that regulates phosphate and vitamin D metabolism. The endothelium of IMAs also contains low-density lipoprotein (LDL) receptors, thrombin, and factor X (Figure 1), all of which are associated with lipid metabolism and the regulation of blood clotting. All these structures are also found on the endothelium of coronary arteries. The endothelium of IMAs is less permeable due to tight connections between ECs. Furthermore, ECs of IMAs produce anti-thrombotic agents and release higher levels of nitric oxide (NO) [1]. Blood clotting in IMAs is prevented by higher levels of PG12 produced than in coronary arteries [4]. The endothelium of IMAs (Figure 1) and coronary arteries contains small leucine-rich proteoglycans (SLRPs) such as biglycan and lumican, as well as large proteoglycans: versican and perlecan. Biglycan interacts with Toll-like receptor 4 (TLR4) to initiate inflammation [5]. The production of biglycan in IMAs is not necessarily higher than in coronary arteries [5] and may not have a specific advantage in regulating inflammation or the production of collagen fibrils and elastin fibers. Further research is required to determine if biglycan can induce autophagy in IMAs. Lumican regulates the signaling pathway of TLR4 [6]. Versican acts as a ligand that binds to multiple receptors and modulates cytokine production [7]. Perlecan regulates the activity of various growth factors, including FGF, PDGF and vascular endothelial growth factor (VEGF) [8].

Kraler et al. [4] reviewed the anatomical and physiological differences between IMAs and coronary arteries and discussed the cellular and molecular properties of IMAs that render them more resistant to atherogenesis. According to the authors, the unique anatomical properties of the endothelium and VSMCs of IMAs, and their ability to resist inflammatory reactions, protect them from intimal growth and atherosclerotic plaque formation. Although the vascular characteristics of IMAs have been well studied, less is known about biochemical reactions and metabolic products involved in preventing the buildup of plaque and AS. We need in-depth studies on the resilience of IMAs to atherogenesis. This may lead to the development of novel medical therapies.

Recent findings have shown that inflammation is driving AS. The NLRP3 inflammasome and its downstream effectors, such as interleukin (IL)-1β and IL−6, play a major role. In this review, the focus is on the composition of IMAs and the effect cytokines have on the physiology of ECs. Our understanding of how IMAs react to inflammation is important, as these arteries are increasingly used in CABG surgery. Numerous studies have referred to the fact that IMAs used in CABG surgery outweigh the use of superior vena cava grafts (SVGs) by triggering minimal atherosclerotic changes and having excellent long-term outcomes and patient survival [1,2,4].

## 2. Synthesis and Functioning of the Endothelium

**Figure 2:**
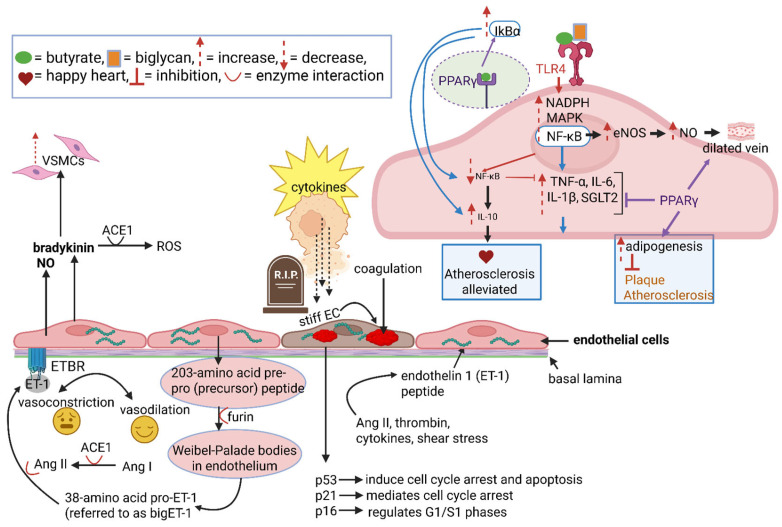
A schematic representation of the endothelium with endothelial cells (ECs), supported by the basal lamina. The rigidity of ECs is shaped by endothelin (ET) peptides, ET-1. The synthesis of ET-1 is regulated by angiotensin II (Ang II), thrombin, cytokines, and shear stress. The inactive 203-amino acid pre-pro (precursor) peptide is cleaved by furin into a 38-amino acid pro-ET-1 (bigET-1). The latter is constitutively released from Weibel-Palade bodies (storage granules) located in the endothelium (drawn separately from the endothelium). ET-1 binds to ETB receptors (ETBRs) on ECs. Ang I is converted to active Ang II by angiotensin-converting enzyme 1 (ACE1). ACE1 degrades bradykinin, and reactive oxygen species (ROS) are released. Compared to coronary arteries, IMAs release higher levels of bradykinin and NO. An increase in cytokines (“cytokine storm”) during a major infection modifies the anatomical and physiological properties of ECs in IMAs and coronary arteries, the endothelium becomes more rigid (stiff), cellular content coagulates, and ECs age (EC senescence). This coincides with the release of regulatory peptides p53, p21, and p16. The binding of butyrate and biglycan to Toll-like receptor 4 (TLR4) stimulates the activity of nuclear factor kappa B (NF-κB), NADPH, and mitogen-activated protein kinase (MAPK), which increases tumor necrosis factor α (TNF-α), interleukin (IL)-6, IL-1β, and protein SGLT2 levels. Binding of butyrate to peroxisome proliferator-activated receptor gamma (PPARγ) suppresses the activity of NF-κB and activates IκBα, a key regulator of NF-κB. PPARγ inhibits the production of TNF-α, IL-6, IL-1β, and protein SGLT2, and stimulates adipogenesis. The schematic representation was constructed using Biorender (Created in Biorender. L.M.T. Dicks. 2025. https://app.biorender.com/illustrations/689b2c9202cb36d3d34c1fbe accessed on 20 August 2025).

The endothelium is composed of a monolayer of ECs that forms the inner cellular lining of arteries, veins, capillaries, and lymphatic vessels [9]. ECs regulate blood flow, prevent blood clotting, and control the exchange of fluids between blood and tissue. ECs are supported by the basal lamina (Figure 2). The rigidity of ECs is shaped by endothelin (ET) peptides. These peptides act as vasoconstrictors but also regulate the production of nitric oxide (NO), a vasodilator [10]. Of the three isoforms (ET-1, ET-2, and ET-3), ET-1 is the most prominent and increases in levels when the endothelium is damaged [11].

The synthesis of ET-1 in IMAs (and coronary arteries) is regulated at the transcriptional level by angiotensin II, thrombin, cytokines, and shear stress (Figure 2) [12]. The inactive 203-amino acid pre-pro (precursor) peptide produced by ECs is cleaved by furin, a calcium-dependent membrane-bound prohormone convertase, into a 38-amino acid pro-ET-1 (referred to as bigET-1; Figure 2). The 38-amino acid peptide is constitutively released from Weibel-Palade bodies (storage granules) located in the endothelium (Figure 2) [13,14]. ET-1 binds to ETB receptors (ETBRs) on ECs (Figure 2) and to ETA receptors (ETARs) on vascular smooth muscle cells (VSMCs) [15]. ET-1 bound to ETAs are primarily associated with vasoconstriction, while ET-1 attached to ETBs regulate vasoconstriction and vasodilation [16]. Prolonged exposure to ET-1 can impair endothelium-dependent relaxation and reduce nitric oxide (NO) bioavailability, potentially damaging the endothelium. It would thus be important to regulate the activity of ET-1 by blocking ETA. This can be achieved with the ETA blockers bosentan, ambrisentan and macitentan, but these are mainly used in the treatment of pulmonary arterial hypertension [17]. Further research is needed to understand the mechanisms by which ET-1 is regulated and vasoconstriction inhibited.

The production of ET-1 by ECs is stimulated by angiotensin II (Figure 2), a key regulator of blood pressure and fluid balance [18]. Angiotensinogen in IMAs and coronary arteries is cleaved by renin (a protease) to form the inactive decameric peptide angiotensin I (Ang I), which is then converted to the active octomeric vasocontrictor angiotensin II (Ang II) by angiotensin-converting enzyme 1 (ACE1) (Figure 2). The latter is present at high levels in the endothelial and adventitial layers of the aorta and plays a major role in regulating blood pressure [19] and fluid balance [20]. ACE1 is also responsible for the degradation of the vasodilator bradykinin, which releases free radicals (ROS) and increases VSMC growth (Figure 2) [21]. Compared to coronary arteries, higher levels of bradykinin and NO (Figure 2) are released by IMAs [22].

The inhibition of ACE1 by lisinopril and captopril (hypertension drugs) results in the remodeling of VSMCs in the myocardium [23] and the suppression of atherosclerosis (AS) [23]. ACE2, a metallopeptidase, present in ECs, pulmonary epithelial cells, and enterocytes (especially in the small intestine), cleaves angiotensin II into the vasodilator angiotensin 1–7, and converts angiotensin I to angiotensin 1–9 [24], thereby decreasing blood pressure. The balance between ACE1 and ACE2 is thus critical in the regulation of blood pressure, electrolyte homeostasis, vascular and cardiac remodeling, and inflammation. Despite our knowledge of the roles of ACE1 and ACE2, more research is required on their regulation.

The activation of RAAS promotes vascular inflammation, neovascularization, oxidative stress, matrix degradation, and impaired efferocytosis, all of which contribute to plaque formation. AngII is a critical mediator of AS. The inhibition of RAAS by ACE inhibitors (ACEi) or AngII receptor blockers (ARB) will reduce blood pressure and stabilize atherosclerotic lesions through anti-inflammatory, anti-fibrotic, and antioxidant mechanisms. Further studies on the inhibition of RAAS are needed to evaluate the therapeutic potential of RAAS inhibition in AS.

## 3. Effect of a “Cytokine Storm” on the Endothelium

A major infection resulting in a drastic increase in cytokines, e.g., as experienced with SARS-CoV-2 infection (the causative agent of COVID-19), modifies the anatomical and physiological properties of ECs in IMAs and coronary arteries [25]. Auguet et al. [26] have shown increased production of cytokines such as TNF-α, interferon-γ and visfatin by endothelin-1 (ET-1) in human VSMCs derived from mammary arteries. More research is needed to understand the prothrombotic and antithrombotic effects of visfatin. The endothelium becomes more rigid (stiff), cellular content coagulates, and ECs start aging (referred to as EC senescence) (Figure 2) [27]. The drastic increase in immune cells elevates ET-1 levels [13] and may lead to long-term cardiovascular complications [27,28]. Patients infected with SARS-CoV-2 have increased plasma levels of interleukin (IL)-1β, IL-6, tumor necrosis factor α (TNF-α), monocyte chemoattractant protein-1 (MCP-1), soluble intercellular adhesion molecule-1 (sICAM-1), and dihydroethidium (DHE) [28]. These changes in immune responses and cell signaling cause severe dysfunction and aging of ECs [27]. Over time, aging and dysfunctional ECs become more inflamed, and coagulated particles instigate cell death that leads to different forms of cardiovascular diseases (CVDs).

An increase in permeability of the endothelium, characteristic of SARS-CoV-2-infections, activates Toll-like receptor 4 (TLR4), which leads to an increase in inflammatory reactions. The role of TLRs is discussed in reviews by Dicks [29,30]. The inflammasome is further activated by the interaction of biglycan with TLR4 and TLR2, and lumican that regulates the TLR4 pathway (Figure 2) [27]. Butyrate, produced by gut bacteria in the large intestinal tract, also activates TLR4 and regulates enzymatic and immunological pathways, together with peroxisome proliferator-activated receptor gamma (PPARγ) (Figure 2), reviewed by Dicks [29]. TLR4 and PPARγ activate nuclear factor kappa B (NF-κB), NADPH, and MAPK [31]. This leads to an increase in endothelial nitric oxidase (eNOS) and NO production (Figure 2) [29]. Protein p22^phox^ and Protein p47^phox^ are key components of NADPH oxidase (NOX) and regulate the production of reactive oxygen species (ROS) [26]. Further research is required to determine if the production of Protein p22^phox^ and Protein p47^phox^ is upregulated in IMAs. If this is the case, it may explain why ROS levels are higher in IMAs. Genes encoding proinflammatory cytokines IL-1β, IL-6, and TNF-α (*IL1B*, *IL6*, and *TNFA,* respectively), and protein SGLT2 that acts as a sodium-glucose cotransporter, are also upregulated (Figure 2) [27]. Thus, cytokines act as potent inducers of SGLT2 expression in coronary ECs of COVID-19 patients through sustained oxidative stress. Proinflammatory cytokines TNF-α, IL-1β, and IL-6 can induce SGLT2 expression in IMAs [32].

Infected and damaged ECs increase the secretion of von Willebrand factor, produce more thrombin (Figure 2), and form more aggregated platelets [28]. The binding of thrombin to thrombin receptors on ECs of IMAs is important in that it mediates the relaxation in IMA [33]. Damaged ECs produce less ACE2 and upregulate mRNA genes associated with aging. Examples of “aging genes” are *TP53*, *CDKN1A*, and *CDKN2A*, encoding proteins p53, p21, and p16 (Figure 2) [27]. Protein p53 activates genes that induce cell cycle arrest and apoptosis (Figure 2) [31]. Protein p21 is a downstream target of p53 and mediates cell cycle arrest (Figure 2) [34]. Protein p16 regulates the transition of cells from the G1 phase (DNA replication and protein synthesis) to the S phase (DNA duplication to form two identical chromatids) (Figure 2) [35]. Other genes upregulated with the infection of ECs are *VCAM1*, *ICAM1*, *SELE*, and *SELP,* encoding the cytoadhesins VCAM-1, ICAM-1, E-selectin, and P-selectin, respectively; *TF* (thrombotic modulator tissue factor, also referred to as F3); *TFPI* (tissue factor pathway inhibitor, TFPI); *THBD* (encoding the protein thrombomodulin); and *PAI-1* (plasminogen activator inhibitor-1, PAI-1) that inhibits fibrinolysis [27]. Differences in the production of p53, p21, p16, VCAM-1, ICAM-1, E-selectin, P-selectin, F3, TFPI, thrombomodulin, and PAI-1 in IMAs compared to coronary arteries may provide interesting information on the aging of IMAs. As mentioned elsewhere, cytokines act as potent inducers of SGLT2 expression in ECs through sustained oxidative stress. The increase in SGLT2 levels in coronary ECs of COVID-19 patients (high cytokine levels) rendered the cells dysfunctional and enhanced aging and inflammation, platelet adhesion, platelet aggregation, and thrombin production [27]. Of interest is that plasma from patients diagnosed with CVDs showed a slight increase in VCAM1, ICAM1, ACE1, AGTR1, CYBA, NCF1, and IL6 compared to healthy patients [27].

The prominent imbalance in the renin–angiotensin–renin-angiotensin-aldosterone system (RAAS), combined with the decline in ACE2, could stimulate the proinflammatory ACE1/Ang II/AT1R axis and trigger a drastic increase in cytokines (“cytokine storm”) [36,37]. Damage to ECs manifests in microvascular thrombosis, capillary leakage, and a decline in oxygen uptake [38,39]. Inflammation of the myocardium correlated with the downregulation of ACE2 [40]. A reduction in ACE2 altered the length of telomeres, characteristic of aging ECs [38,41]. In severe SARS-CoV-2 infections, ECs are permanently damaged, resulting in excessive vasoconstriction, inflammation, and thrombosis [42,43]. If damaged beyond repair, ECs are substantially modified [25], and arteries may become chronically inflamed [44]. In this review, SARS-CoV-2 infection is used as an example that induces a “cytokine srtorm”. For further information on the correlation between COVID-19 and atherosclerosis, the reader is referred to papers published by Jalili et al. [44], Saeed et al. [45], Nishiga et al. [46], and Grzegorowska and Lorkowski [47].

Inflammatory reactions are also generated by commensal gut microbiota. Examples are succinate that inhibits the enzyme prolyl hydroxylase (PHD) involved in the regulation of hypoxia, fumarate that controls the release of mitochondrial DNA and RNA [48], short-chain fatty acids (SCFAs) that interact with Toll-like receptor 4 (TLR4) but also platelet glycoprotein 4 (CD36), and G protein receptors (GPRs) [49], and lipopolysaccharides (LPS) [50]. Toxins produced by gut microbiota weaken the bond between tight junction proteins in the intestinal epithelium, allowing bacteria and their components to enter the bloodstream and trigger inflammatory responses [50]. As a result of these changes, the levels of AGEs (advanced glycation end products) and ROS increase [51,52,53]. With the increase in inflammation, drastic changes in intestinal permeability are initiated [54], leading to a cascade of reactions [29]. An increase in oxidative stress leads to the production of ox-LDL, which is taken up by macrophages and transformed into lipid-rich foam cells. The foam cells instigate the formation of arterial plaques, reviewed by Dicks [29] and Matsuura et al. [55]. T- and B-lymphocytes are also involved in the development of plaque and atherosclerosis (AS) [56]. CD4+ T-cells differentiate into helper T (Th) cells. Th1 cells express interferon-γ (INF-γ) to promote AS, whilst Th2 cells produce IL-4, IL-5, and IL-13 that neutralize INF-γ [57,58,59]. In summary, activation of TLR4 stimulates the nuclear factor kappa B (NF-κB) and mitogen-activated protein kinase (MAPK) pathways, signaling an increase in cytokines, chemokines, eNOS, and Treg cells (Figure 2). The overstimulation of NF-κB may induce the transformation of ECs into mesenchymal cells in the aorta, leading to calcification and stenosis (Figure 2) [60]. Activated peroxisome proliferator-activated receptor gamma (PPARγ) acts as a dilating agent, regulating hypertension [61] and stimulates adipogenesis to prevent plaque formation (Figure 2) [29].

VSMCs are maintained by Notch 3, which regulates gene transcriptions, intracellular communication, neural development, and the binding of epidermal growth factor (EGF) to EGF receptors (EGFRs) [62]. Trimethylamine-N-oxide (TMAO), produced by gut microbiota, activates Nod-like receptor protein 3 (NLRP3) and triggers platelet formation [29,63,64]. Information on NLRP3 production in IMAs is limited and warrants further investigation to understand why IMAs are less prone to platelet formation. Gut bacteria such as *Escherichia* and *Klebsiella* are known to increase TMAO levels [65].

IMAs have small branches that supply the chest wall and breast tissue. Small blood vessels, specifically the vasa vasorum and lymphatic vessels within the arterial wall, limit the trafficking of immune cells and reduce plaque buildup [66]. In atherosclerosis, these vessels can proliferate (neovascularization) and contribute to inflammation within the artery. Paraoxonase 2 (PON2), highly expressed in VSMCs of IMAs, suppresses the production of reactive oxygen species (ROS) and protects mitochondria against oxidative stress (reviewed by Dicks [30]). PON1 acts as an antioxidative, anti-inflammatory, antiapoptotic, vasodilative, and antithrombotic agent [67]. PON2 (and PON3) hydrolyzes the bacterial quorum-sensing molecule N-3-oxododecanoyl homoserine lactone (3OC12) [68], preventing it from interacting with immune defense systems, especially the entry of polymorphonuclear neutrophils (PMNs) into infected sites [69]. This reduces inflammation and oxidative stress. An in-depth understanding of anti-inflammatory pathways could identify future targets with promising novel therapeutic possibilities, including anticytokine therapy. A research area that receives less attention is the cytokine activation of intracellular signaling pathways in ECs and its effect on gene expression and cellular behavior. This may lead to the discovery of drugs targeting specific cytokine receptors on ECs, potentially modulating their activity to prevent or reverse endothelial dysfunction.

## 4. Alleviation of Endothelial Injuries

Damaged endothelial cells are unable to regulate the production of NO, which is important in the prevention of thrombosis and chronic inflammatory reactions within the arterial wall [70]. Small ubiquitin-like modifier (SUMO) proteins that alter the function and stability of proteins also influence interactions with other proteins. Zhang et al. [71] have shown that NO, produced by ECs, activated transcription factor 3 (ATF3). The authors noted that the expressions of ATF3 and SUMO-1 were drastically enhanced in angiotensin II (Ang II)-induced ECs. Concluded from these findings, AngII accelerated the SUMOylation of ATF3 by lowering the production of NO. It may thus be concluded that SUMO regulates the release of inflammatory cytokines and the expression of adherence molecules. This was indeed reported by Liu et al. [72]. The authors have shown that SUMOlyation modified the NF-κB and TLR pathways. Qiu et al. [73] reported that the transcription factor GATA-binding protein 2 (GATA2) activated ECs. GATA2 plays a crucial role in hematopoiesis and endocrine cells [74]. Mutations in the *GATA2* gene can lead to a variety of disorders, known as GATA2 deficiency syndrome [74]. A deficiency in *SENP1* enhanced SUMOylation of GATA2 and the production of IκBα (inhibitor of NF-κB) [73]. This may suppress inflammation and may be of value in the treatment of inflammatory reactions associated with AS. Research on the SUMOlyation in IMAs is limited.

Damage to endothelial cells activates the NLRP3 inflammasome and accelerates the development of AS by activating interleukin (IL)-1β and IL-18 [75]. The incorrect SUMOylation of NLRP3 enhanced the release of IL-1β. However, defective SUMO-specific proteases SENP7 prevented the release of IL-1β [76]. The SUMOylation of NLRP3 may be a novel approach to treat AS.

Pigment Epithelium-derived Factor (PEDF), also known as serine protease inhibitor (SERPIN) F1 [77,78], is structurally homologous to other SERPIN family proteins, but does not inhibit proteases [79] and is expressed in ECs, cardiomyocytes, fibroblasts, macrophages, and adipocytes [80]. PEDF suppresses inflammatory cytokines such as IL-1β, IL-6, TNF-α, and IL-17A [81], Th17 cells, the phosphorylation of the stress-activated p38, and c-jun N-terminal protein kinases (JNK/SAPK) [80,82]. PEDF also inhibits angiotensin II (Ang II)-induced EC activation by suppressing NOX-mediated ROS production [78]. Yamagishi et al. [78] reported that the anti-oxidative properties of PEDF block tumor necrosis factor (TNF)-induced EC activation. PEDF, upregulated by D-4F, an apolipoprotein, reduces ox-LDL-induced damage to ECs [80] and inhibits the Wnt/β-catenin pathway [83]. Despite extensive research on PEDF and IMAs, little is known about its role in IMAs.

SUMOylation regulates the expression of the scavenger receptor Snmp1 (required for cholesterol uptake in steroidogenic tissues), and Sterol Regulatory Element Binding Proteins (SREBPs) that coordinate lipid homeostasis [84]. This is a carefully controlled process, as cholesterol is required for the synthesis of steroid hormones, bile acids, oxysterols, and vitamin D. Low cholesterol levels are associated with neuropsychiatric disorders and the Smith–Lemli–Opitz syndrome (caused by a mutation in the DHCR7 gene, which prevents the body from making cholesterol) [85]. Excessive cholesterol is associated with cardiovascular diseases and cancer [86].

SUMO proteins are highly expressed by myocardial cells and are closely associated with myocardial infarction (MI), ischemia, myocardial necrosis, heart failure, and cardiac arrest [87,88,89]. The dysfunctioning of ECs, dyslipidemia, and abnormalities in VSMC proliferation were identified as primary pathological events in the initiation and progression of AS [90]. It is thus important to understand the molecular mechanisms behind SUMOylation. This requires an in-depth study on SUMOylation and deSUMOlyation. For further background on SENPs and deSUMOylation, the reader is referred to the reviews by Liu et al. [72], Jia et al. [91], Brackett et al. [92] and Zhao et al. [89].

Recent findings have shown that SCFAs and BCFAs, produced by gut microbiota, inactivate deSUMOylases (isopeptidases) responsible for the destruction of SUMO peptides in the human gastrointestinal tarct (GIT) [93]. This results in the hyperSUMOylation of peptides and prevents their transport across cell membranes [94]. Little is known about the influence of gut microbiota on SUMOlyation and deSUMOlyation.

## 5. IMA Graft Patency and Clinical Implications

The first reported use of IMAs for coronary artery bypass grafting (CABG) in humans was in 1961 [95]. The technique was only fully appreciated in 1966 when Favaloro introduced bilateral IMA implants, published the following year [96]. This initiated the practice of using IMAs as conduits for direct myocardial revascularization. For more information on the development of CABG, the reader is referred to the review by Squiers and Mack [97]. Today, IMA grafting is considered the “gold standard” in CABG [98]. Patency rates of more than 90% have been reported over 5 years [99,100,101]. Kitamura et al. [102] reported patency rates of 100% at the early and late postoperative periods in pediatric patients 5 to 13 years after surgery. Findings from this study indicated that IMA grafts may serve as “live” (growing) conduits and that they are suited for pediatric CABG. Between 85% and 95% of IMA grafts remained open 7 to 10 years after surgery [98]. IMA-to-left anterior descending (LAD) coronary artery grafting is more durable [103]. Patients with IMA grafts required fewer follow-up interventions [104]. Risk factors associated with IMA grafts are rare but may include anastomotic leakage, abnormal narrowing of the connected area, microbial infection, and bleeding [98]. As with all CABG, age, diabetes, and other comorbidities can influence the long-term success of IMA grafting.

Clinical implications using IMAs as CABGs compared to SVGs include improved long-term survival, reduced risk of myocardial infarction and angina, and a lower risk of repeat revascularization [98]. The resistance of IMAs to develop AS and their ability to maintain potency for extended periods are key factors contributing to these benefits. The benefits of IMA grafting extend to various patient subgroups, including those with normal or impaired ventricular function, men and women, and patients of different age groups [105].

## 6. Conclusions

Although IMAs and coronary arteries share many features, IMAs are more rigid due to the neointima protective layer located between the intima and internal elastic lamina. Damaged ECs of IMAs are protected from blood clotting by an increase in prostacyclin (PGI2), a potent inhibitor of platelet aggregation. Angiotensin II, which binds to receptors on VSMCs, is a powerful vasoconstrictor that plays a key role in regulating blood pressure and fluid balance. Bradykinin (a vasodilator) is degraded by ACE1, preventing overactivity. ACE inhibitors, e.g., used to treat hypertension, increase bradykinin levels. Higher levels of bradykinin and NO are released from IMAs than from coronary arteries. Inhibiting or suppressing the activity of ACE1, as in IMAs, also remodels VSMCs in the myocardium, and suppresses AS. ACE2, on the other hand, is also present in ECs, cleaves Ang II, disabling its function as a vasoconstrictor, thus decreasing blood pressure. The SERPIN PEDF inhibits AngII and suppresses the activities of ECs, including the activity of NOX and the production of ROS. PEDF, upregulated by apolipoprotein D-F4, suppresses the production of oxidized low-density lipoprotein (ox-LDL) and blocks TNF-induced EC activation. Damaged ECs produce less ACE2 and upregulate mRNA genes encoding proteins involved in apoptosis (protein p53), cell cycle arrest (protein P21), and cell growth, e.g., switching from the G1 phase to S1 phase (DNA duplication). A decline in ACE2, together with an imbalanced RAAS, could stimulate the proinflammatory ACE1/AngII/AT1R axis and trigger a drastic increase in cytokines (“cytokine storm”). Maintaining a balance between ACE1 and ACE2 is important, as they regulate electrolytes, blood pressure, vascular and cardiac cells, and inflammation. It is equally important to maintain a balanced gut microbiome. A gut microbiome in dysbiosis may produce excessive succinate, fumarate, short-chain fatty acids (SCFAs), lipopolysaccharides (LPS), and toxins that alter the permeability of the intestinal epithelium and increase cytokine levels. Little is known about the influence of gut microbiota on SUMOylation and deSUMOylation. Further research is required, as SUMO proteins alter the NF-κB and TLR pathways that regulate the release of inflammatory cytokines and NO production. This will affect ECs in the intima of IMAs and coronary arteries. Further research on the mechanical resistance of IMAs is required to understand the effect of shear stress forces, factors affecting the stability of tight junctions in ECs that may influence elasticity, conditions that would maintain an increase in the production of NO, and factors suppressing selectins and other adhesion molecules. We need to incorporate modern technological advances, such as multi-omics and systems biology approaches, to identify novel biochemical/physiological reactions and pathways that are involved in rendering IMAs resilient to atherogenesis. We also need to understand how different molecules and structures interact with each other. Once we fully understand which key mechanisms are involved in atherogenesis, we may be in a better position to develop novel anti-atherosclerotic treatment strategies and discover molecular targets to diagnose AS and CVDs at an early stage.
